# Beyond the Motor Cortex: Thalamic Iron Deposition Accounts for Disease Severity in Amyotrophic Lateral Sclerosis

**DOI:** 10.3389/fneur.2022.791300

**Published:** 2022-02-24

**Authors:** Qianwen Li, Wenjia Zhu, Xinmei Wen, Zhenxiang Zang, Yuwei Da, Jie Lu

**Affiliations:** ^1^Department of Radiology and Nuclear Medicine, Xuanwu Hospital, Capital Medical University, Beijing, China; ^2^Beijing Key Laboratory of Magnetic Resonance Imaging and Brain Informatics, Beijing, China; ^3^Department of Neurology, Xuanwu Hospital, Capital Medical University, Beijing, China

**Keywords:** amyotrophic lateral sclerosis (ALS), iron deposition, gray matter atrophy, motor cortex, thalamus

## Abstract

**Objective:**

Previous studies have reliably identified iron deposition in the motor cortex as potential pathogenesis of amyotrophic lateral sclerosis (ALS). Here, we intended to investigate iron deposition, gray matter (GM) atrophy, and their associations with disease severity in the motor cortex and the thalamus in patients with ALS.

**Methods:**

A total of 34 patients with ALS (age 51.31 ± 8.24 years, 23 males) and 34 nonneurological controls (age 50.96 ± 9.35 years, 19 males) were enrolled between 2018 and 2020. The Revised ALS Functional Rating Scale (ALSFRS-R) and the Penn upper motor neuron (UMN) score were measured. MRI data included quantitative susceptibility mapping (QSM) for iron deposition and three-dimensional (3D) T1 for gray matter volume. After a between-group comparison, Pearson's correlation coefficient was used for identifying correlations of iron deposition, GM volume, and clinical measurements.

**Results:**

The two-sample *t*-tests revealed increased iron deposition in the left precentral gyrus (peak voxel *T* = 4.78, *P*_SVC_ = 0.03) and the thalamus (peak voxel: right: *T* = 6.38, *P*_SVC_ < 0.001; left: *T* = 4.64, *P*_SVC_ = 0.02) in patients with ALS. GM volume of the precentral gyrus (*T* = −2.42, *P* = 0.02) and the bilateral thalamus (*T* = −4.10, *P* < 0.001) were reduced. Negative correlations were found between the increased QSM values and the decreased GM volume (*P* < 0.04, one-tailed) in patients with ALS. Iron deposition in the left precentral gyrus was positively correlated with the UMN score (*R* = 0.40, *P* = 0.02) and the GM volume was negatively correlated with the UMN score (*R* = −0.48, *P* = 0.004). Negative correlation between thalamic iron deposition and the ALSFRS-R (*R* = −0.36, *P* = 0.04) score was observed.

**Discussion:**

Iron deposition in the thalamus, in addition to the motor cortex, is accompanied by GM atrophy and is associated with disease severity in patients with ALS, indicating that the thalamus is also a pathological region in patients with ALS.

## Background

Amyotrophic lateral sclerosis (ALS) is a progressive neurodegenerative disorder characterized by impairments of motor neurons in the motor cortex, brainstem, and spinal cord (1). Nowadays, increasing observations of extramotor impairments were reported in ALS studies (2, 3). Particularly, as one of the major subcortical areas that modulate information *via* the thalamocortical loop, the thalamus is critical for the investigation of the pathological mechanism of ALS (4, 5).

Studies over the past decades have shown gray matter (GM) atrophy in the motor cortex and the thalamus in patients with ALS (6, 7). Patients with ALS exhibited GM volume reduction in the motor cortex and the thalamus as shown in a longitudinal study (8). In a recent large cohort MRI-based study, Chipika and colleagues have shown atrophy of thalamic nuclei in both the chromosome 9 open reading frame 72 (C9orf72) carrier and noncarrier patients with ALS, demonstrating thalamus as a potential pathological area in the brain (9).

Furthermore, accumulating evidence suggested that iron deposition in the brain might have an important role in the pathogenesis of ALS (10, 11). A large amount of *in-vivo* MRI studies had reported iron deposition in the motor cortex (10, 12, 13), which was in line with the postmortem study (10). Further, iron deposition in the brain was positively associated with the upper motor neuron (UMN) score of the corresponding limb (13). While postmortem examination (14) had shown iron deposition in the thalamus, *in-vivo* evidence showing how iron content differentiates between patients with ALS and nonneurological controls (NCs) in the thalamus is still lacking. Besides, little, if not nothing, is known regarding the association between thalamus iron deposition, GM atrophy, and clinical measurements.

On the cellular and molecular level, excessive iron deposition may lead to oxidative stress-associated neural apoptosis (15). On the macroscopic scale, cortical iron deposition in the precentral gyrus was correlated with GM atrophy in patients with multiple sclerosis (16). Suggestively, therefore, we hypothesize that the accumulation of iron content would accompany progressive GM atrophy in the motor cortex and the thalamus in patients with ALS. Investigation of their associations may provide new insights for underlying the mechanism of ALS.

Conclusively, we have two aims in this study. First, we intended to assess iron deposition and GM atrophy in the motor cortex and the thalamus in patients with ALS by using quantitative susceptibility mapping (QSM) and three-dimensional (3D) T1 structural MRI modalities. Second, we intended to investigate how iron deposition and GM atrophy correlate with clinical neurological measurements.

## Materials and Methods

### Study Participants

This study was approved by the Ethic Committees of Xuanwu Hospital, Capital Medical University. We collected 40 patients with ALS and matched 42 non-NCs among volunteers and nonblood relatives between December 2018 and January 2020. Patients with ALS were defined as “probable” or “definite” according to the revised El Escorial criteria (17). The NCs were recruited in Xuanwu Hospital with similar age and sex with the ALS patients. The controls did not show any signs of movement problem during the interview. Both the patients with ALS and the NCs were considered cognitively normal *via* the neurologists during the interview. All the participants were free of substance drug abuse, traumatic brain injury, cerebrovascular events, neuroinflammation, neoplastic conditions, and were able to participate in MRI scanning (e.g., without metallic plant-in devices and severe disability). After the MRI scanning, one patient with ALS was excluded due to claustrophobia, and three patients with ALS and two controls were excluded due to severe motion artifacts. No space-occupying lesions were found in patients.

We recorded clinical characteristics, including the disease duration (DD) (number of months from the ALS symptoms onset to the MRI examination), the Revised ALS Functional Rating Scale (ALSFRS-R) (18), and the Penn UMN score (19). We then defined the disease progression rate (DPR) delta-FRS, which was calculated by using the formula: DPR = (48-ALSFRS-R)/DD (20).

### Image Acquisition

The imaging data were collected on a 3T scanner (MR750, GE Healthcare Medical Systems, Chicago, Illinois, USA) equipped with an 8-channel head coil at the Xuanwu Hospital, Capital Medical University, Beijing, China. The standard MRI protocol included a multi-echo gradient echo (GRE) sequence and one 3D T1-weighted acquisition [3D brain volume imaging (BRAVO)]. The MR protocol is shown as below: GRE acquisition parameters: 12 echoes with TE1 = 2.1 ms and Δecho time (TE) = 2.4 ms, repetition time (TR) = 31.1 ms, flip angle = 20°, FOV = 256 mm × 256 mm, slice thickness = 1 mm, matrix = 256 × 256, and 6.5-min acquisition time. 3D BRAVO acquisition parameters: TR = 6.66 ms, TE = 2.93 ms, inversion time (TI) = 450 ms, flip angle = 12°, field of view (FOV) = 256 mm × 256 mm, slice thickness = 1 mm, matrix = 256 × 256, and 4-min acquisition time.

### Image Processing

#### Quantitative Susceptibility Mapping

Quantitative susceptibility mapping is a technique that calculates the quantitative magnetic susceptibility values of tissues by deconvolving MRI phase signals with models of magnetic susceptibility.

The reconstruction of susceptibility MRI was performed by using STI suite version 3.0 (https://people.eecs.berkeley.edu/~chunlei.liu/software.html). In summary, phase image was combined with magnitude for new phase calculation. Then, the magnitude images were used for segmentation by using the brain extraction tool (BET) in FSL to generate a brain mask (22). The new phase images were then unwrapped with the Laplacian method (23) and the background phase was removed by using the variable-kernel sophisticated harmonic artifact reduction for phase data (V-SHARP) filtering method. Finally, the susceptibility map was generated by using the STQR-QSM (streaking artifacts reduction QSM) algorithm (24). The susceptibility map contains the QSM values [parts per million (ppm)] in each voxel, which correlated strongly with the chemically determined iron concentration in the GM structures as shown in a previous postmortem study (25).

For spatial normalization purposes, we first registered the individual susceptibility maps onto the 3D T1 images. Next, the T1 images were segmented into six tissue maps and normalized by using Diffeomorphic Anatomical Registration Through Exponentiated Lie Algebra (DARTEL) algorithm (26) into the standard Montreal Neurological Institute (MNI) template. The deformation field was generated as the normalization parameters for the T1 images, which was later directly applied to the coregistered individual susceptibility maps. Images from eight participants were excluded due to inaccurate normalization (mismatch between template and QSM image of an individual, shown in Supplementary Figure S1 as an example), leaving 34 patients with ALS and 34 NCs into group analyses.

#### Voxel-Based Morphometry (VBM) Analysis

T1 data were analyzed by using the standard VBM approach to calculate the GM volume. In detail, T1-weighted structural images were preprocessed by using the CAT12 toolbox (http://www.neuro.uni-jena.de/cat/). All the structural images were segmented into GM, white matter (WM), and cerebrospinal fluid (CSF). The GM images were normalized to the MNI space by using high-dimensional DARTEL normalization. Images were modulated *via* the Jacobian transformation to ensure that relative GM volumes were well preserved following spatial normalization. Prior to statistical analysis, the normalized modulated GM images were smoothed by using a 4-mm full width at half maximum (FWHM) Gaussian kernel.

### Statistical Analysis

Comparison of QSM between the ALS and control group was conducted in SPM12 in a voxel-wise manner. We used small volume correction (SVC) to account for multiple comparison issues within the precentral gyrus and the thalamus. Clusters with (1) peak voxel surviving *P* < 0.05 SVC within the corresponding brain area [20 mm sphere around the peak voxel overlapped with the corresponding automated anatomical labeling (AAL) regions (27)] and (2) with over 100 mm^3^ (100 voxels) surviving an arbitrary threshold (*P* < 0.005 uncorrected) were considered significant. We used the latter arbitrary criteria to ensure that the size of the significant clusters was not too small.

The regions that showed significant changes of QSM value were further used as a region of interest (ROIs) for extracting the mean GM volumes of brain structures including the precentral gyrus and the thalamus. After the QSM comparison, the GM volumes of the precentral gyrus and the thalamus were extracted by using the clusters that showed an increased iron deposition. We used SPSS software (IBM SPSS Statistics, version 25.0.0.1, IBM Incorporation, Armonk, New York, USA) for *post-hoc* analyses including comparisons of GM volume, associations with the QSM values as well as clinical measurements. The Pearson's correlation coefficient was used to assess the correlation between the QSM value and GM volume, QSM value, GM volume, and their associations with clinical measurements. Sex difference between the ALS and NCs groups was assessed with the chi-squared test. For analyses conducted in SPSS, results were considered significant if *P* < 0.05.

## Results

### Clinical Data

A total of 34 patients with ALS and 34 age- and sex-matched NCs were finally included in our analyses. Five of the patients with ALS were bulbar onset. Detailed demographic information of all the subjects is shown in Table 1. There were no significant differences in age (*P* = 0.56) and sex (*P* = 0.32) between the two groups.

**Table 1:** The demographic and clinical profile of study participants.

**Characteristic**	**ALS patients**	**Non-neurological controls**	***P* values**
Number	34 (40 recruited)	34 (42 recruited)	
Age	51.31 ± 8.24 (SD)	50.96 ± 9.35 (SD)	0.56
Sex (male)	23 (67.6%)	19 (55.9%)	0.32
ALSFRS-R	40.06 ± 4.15 (SD), [29–45]	NA	
DD (month)	16.72 ± 15.77 (SD), [2–84]	NA	
DPR (delftaFRS)	0.9 ± 0.95 (SD), [0.04–3.75]	NA	
UMN score	9.88 ± 6.04 (SD), [0–16]	NA	

*ALSFRS-R, Revised Amyotrophic Lateral Sclerosis Functional Rating Scale; DD, disease duration; DPR, disease progression rate; UMN score, upper motor neuron score*.

### Imaging Data

#### Increased Iron Deposition in the Left Precentral Gyrus and the Bilateral Thalamus

**Figure 1:**
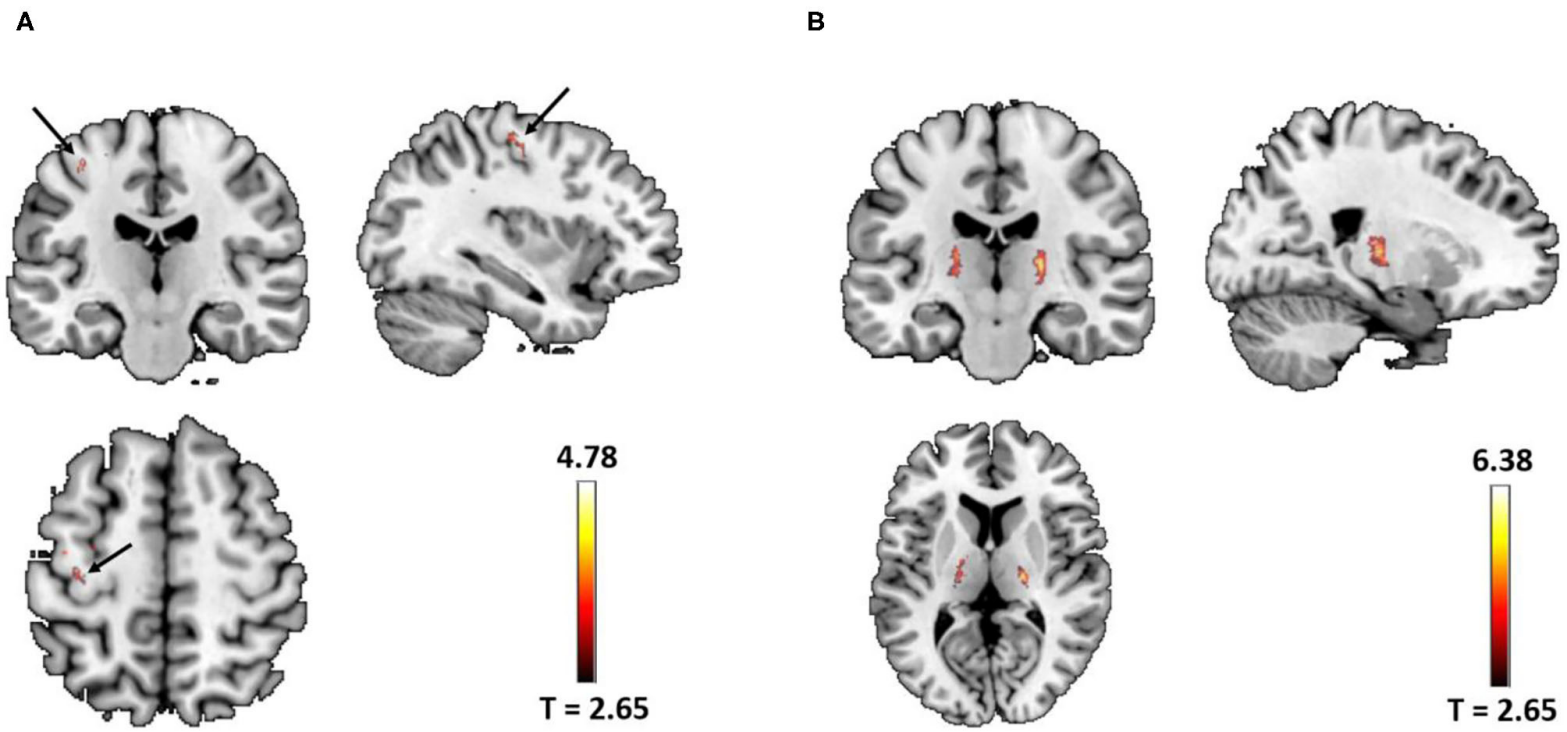
**(A)** Significant increased quantitative susceptibility mapping (QSM) values were found in the left precentral gyrus (arrow) in patients with amyotrophic lateral sclerosis (ALS) (peak voxel *P*_SVC_ = 0.03). **(B)** Significant increased QSM values were found in the bilateral thalamus in patients with ALS (left *P*_SVC_ = 0.02; right *P*_SVC_ < 0.001). Images were shown with a *P* < 0.005 uncorrected thresholds and masked with the left precentral and the thalamus from the AAL atlas. In total, there were 220 voxels and 1,674 voxels surviving *P*_uncorrected_ < 0.005 thresholds in the left precentral gyrus and the bilateral thalamus, respectively.

We focused only on the precentral gyrus and the thalamus. Compared with NCs, the two-sample *t*-test revealed the significant increased QSM values in the left precentral gyrus (peak MNI coordinate: x = −40, y = −24, z = 59, peak *T* value = 4.78, *P*_SVC_ = 0.03, 220 voxels with *P*_uncorrected_ < 0.005) and the thalamus (right: peak MNI coordinate: x = 20, y = −17, z = 7, peak T value = 6.38, *P*_SVC_ < 0.001; left: peak MNI coordinate: x = −13, y = −5, z = 7, peak T value = 4.64, *P*_SVC_ = 0.02, 1,674 voxels with P_uncorrected_ < 0.005) in patients with ALS (Figure 1). No significant increased QSM values were found in the right precentral gyrus (82 voxels survived *P* < 0.005 uncorrected threshold). The peak voxel did not reach small volume correction significance, with *P*_corrected_ > 0.9, *T* = 3.89). The QSM value of the two groups was normally distributed (Lilliefors test, *P* > 0.31). Whole-brain increasing QSM distribution was given in Supplementary Figure S2.

#### Gray Matter Atrophy in the Left Precentral Gyrus and the Bilateral Thalamus

**Figure 2:**
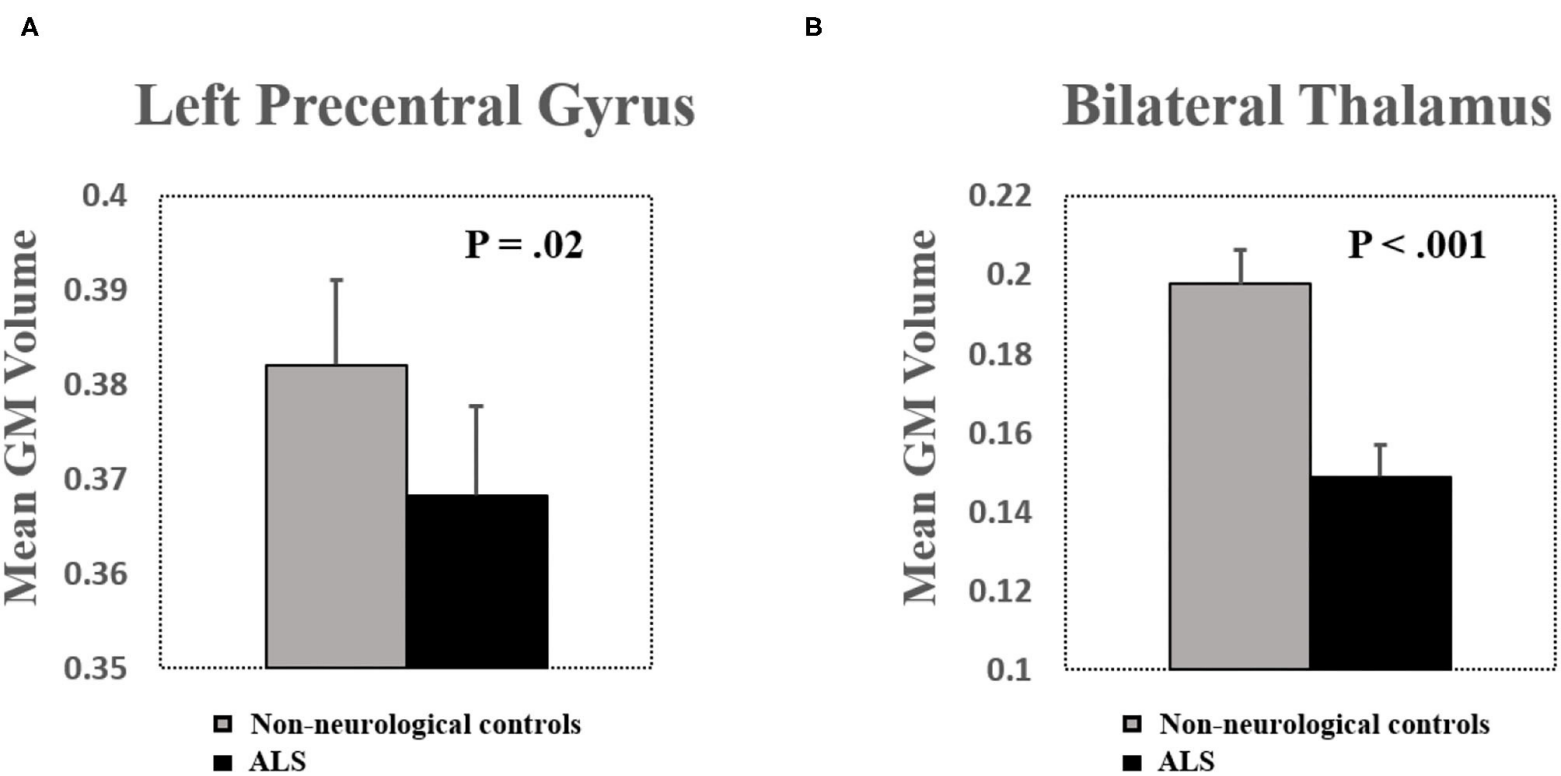
**(A)** Significant brain volume atrophy was found in the left precentral gyrus in patients with ALS. **(B)** Significant brain volume atrophy was found in the bilateral thalamus in patients with ALS. Bar plots show mean gray matter (GM) volume after controlling for total intracranial volume (TIV) as a covariate of noninterest. Error bars show SEs.

Mean GM volume of the left precentral gyrus and the bilateral thalamus was extracted from the clusters shown in Figure 1 (*P*_uncorrected_ < 0.005). Decreased GM volume was found in the precentral gyrus (*T* = −2.42, *P* = 0.02) and the bilateral thalamus (*T* = −4.10, *P* < 0.001) in patients with ALS (Figure 2). Total intracranial volume (TIV) was controlled as a covariate of noninterest. The GM volume of the two groups was normally distributed (Lilliefors test, *P* > 0.14). Whole-brain GM reduction distribution was given in Supplementary Figure S3.

#### Association Between Iron Deposition and GM Atrophy

**Figure 3:**
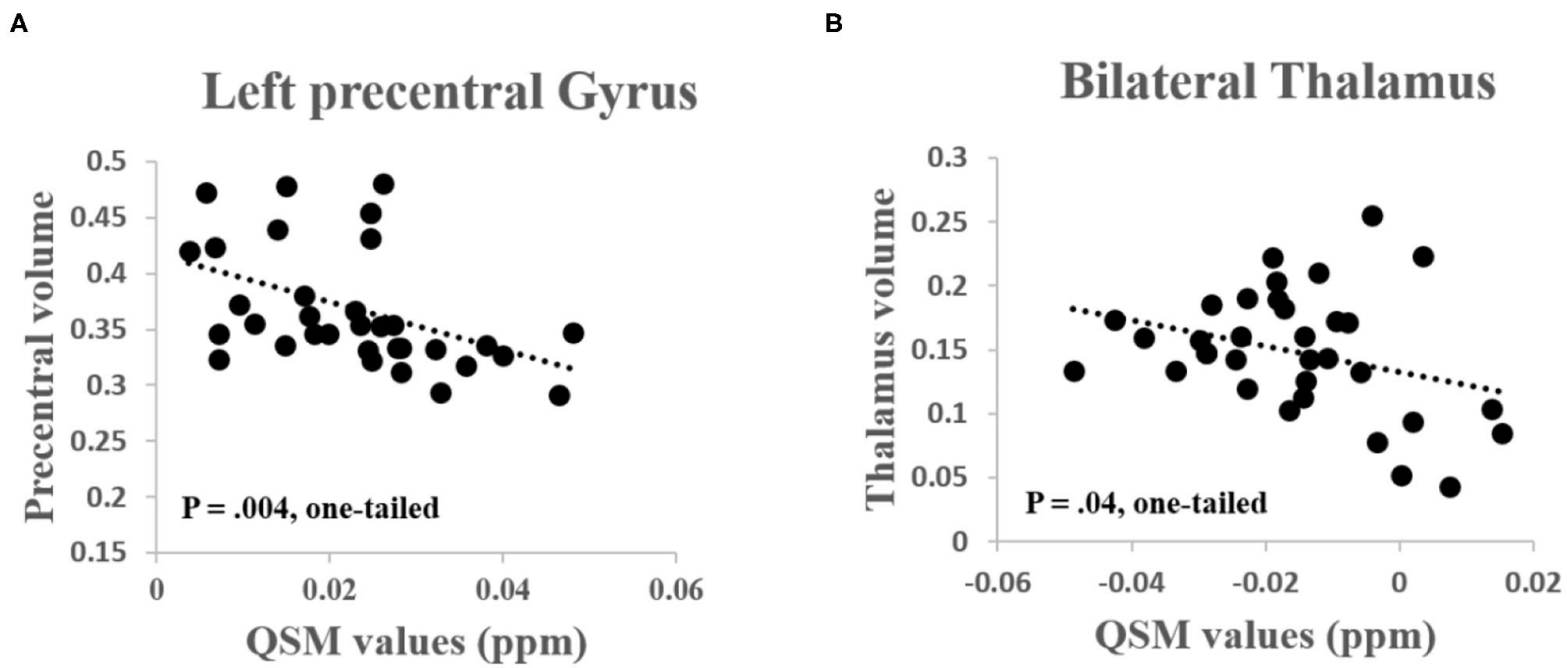
**(A)** Significant negative correlation was found between the QSM values and GM volume in the left precentral gyrus (*R* = −0.44, *P* = 0.004, one-tailed) and **(B)** Bilateral thalamus (*R* = −0.32, *P* = 0.04, one-tailed) in patients with ALS.

Since patients with ALS simultaneously exhibited increased iron deposition and decreased GM volume, we, therefore, specifically investigated the negative correlation between the two phenotypes. As shown in the results, the QSM values were significantly negatively correlated with the GM volume. In details, the QSM values were negatively correlated with GM volume in the left precentral gyrus (*R* = −0.44, *P* = 0.004, one-tailed) and the bilateral thalamus (*R* = −0.32, *P* = 0.04, one-tailed) in patients with ALS (Figure 3). The QSM values were neither correlated with GM volume in the left precentral gyrus (*R* = −0.17, *P* > 0.16, one-tailed) nor in the bilateral thalamus (*R* = −0.19, *P* > 0.14, one-tailed) in the control group.

#### Clinical Associations

**Figure 4:**
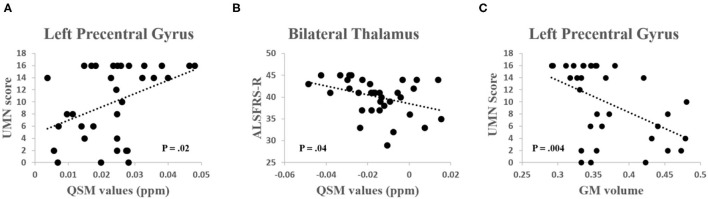
**(A)** A significant positive correlation was found between the QSM values in the left precentral gyrus and the upper motor neuron (UMN) score (*R* = 0.40, *P* = 0.02). **(B)** A significant negative correlation was found between the QSM values in the thalamus and the Revised ALS Functional Rating Scale (ALSFRS-R) score (*R* = −0.36, *P* = 0.04). **(C)** A significant negative correlation was found between the GM volume in the left precentral gyrus and the UMN score (*R* = −0.48, *P* = 0.004).

Quantitative susceptibility mapping value of the left precentral gyrus was significantly positively correlated with the UMN score (*R* = 0.40, *P* = 0.02) and the QSM value of the bilateral thalamus was significantly negatively correlated with the ALSFRS-R (*R* = −0.36, *P* = 0.04) (Figures 4A,B). GM volume of the left precentral gyrus was negatively correlated with the UMN score (*R* = −0.48, *P* = 0.004) (Figure 4C). No other significant correlations were found between the QSM value, GM volume, and other clinical characteristics (including DD, ALSFRS-R, and the DPR, all *P* > 0.15).

#### Age and Sex Influence

We performed correlation analyses on age and QSM/GM values in both the patients and control group and found no significant correlations in any group (lowest *P* = 0.31). We then controlled age as a covariate of noninterest and still found significant correlations between QSM and GM (*P* = 0.004 for precentral gyrus and *P* = 0.028 for thalamus, one-tailed). In addition, there were no significant differences between male and female on QSM/GM values (lowest *P* = 0.33) for either group.

## Discussion

Our primary goal was to investigate iron deposition and GM atrophy as well as their association in the motor cortex and thalamus in patients with ALS. We found an increased iron deposition and decreased GM volume in the motor cortex and the thalamus *via* QSM and VBM approaches, respectively. In patients with ALS, the increased iron deposition was negatively correlated with GM volume. Furthermore, iron deposition in the motor cortex was positively correlated with the UMN score, while iron deposition in the thalamus was negatively correlated with the ALSFRS-R score.

Since impairments of the motor cortex are the hallmark feature, most ALS imaging studies focus on evaluating structural and functional alterations of this region. As a result, GM volume reduction and iron deposition in the motor cortex have been consistently identified and replicated (6, 7, 10). In this study, as expected, we detected a higher QSM value in a cluster adjacent to the “hand-knob” of the left precentral gyrus. We, in addition, found decreased GM volume in the same cluster. Similarly, we detected increased QSM value and decreased GM volume that largely overlapped with the ventral lateral and posterior nucleuses of the thalamus—areas that were functionally connected with the motor and somatosensory cortex (28, 29). Higher QSM value in the thalamus was highly in line with a postmortem study that revealed iron deposition in the thalamus (14). The increase of iron deposition in the brain is consistent with the observations from postmortem studies that reported intraneuronal TAR DNA-binding protein-43 (TDP-43) immunoreactivity and ferritin-positive microglia and macrophages. That being said, increased QSM in patients with ALS may indicate oxidative stress–induced cell death and, possibly, phosphorylated 43kDa TAR DNA-binding protein (pTDP-43) accumulation (30). Our results support the idea of motor cortical and thalamic iron accumulation as pathological phenotypes in patients with ALS.

Increased iron deposition may result in the production of toxic free radicals, which can subsequently cause oxidative stress to neurons that may attenuate neuronal function (15, 31, 32). Indicatively, excessive iron accumulation may reflect neurodegeneration. As shown in a clinical trial, iron chelation such as deferiprone can preserve behavioral motor functions and reduce iron concentration levels in the spinal cord, medulla oblongata, and motor cortex (33). This study supported the idea that iron deposition may be a novel therapeutic target for treatment.

In this study, we found that the QSM value was significantly correlated with the GM volume in the precentral gyrus and the thalamus in patients with ALS but not controls. Although evidence was lacking in ALS studies to show iron deposition-GM atrophy coupling, a comparable result was presented in the multiple sclerosis (16) study where researchers found a significant association between iron deposition and reduction of GM volume in the left precentral gyrus. In this study, the significant relationship between iron deposition and GM volume in the precentral gyrus and the thalamus suggested that excessive iron content may contribute to brain structural atrophy. Since a large proportion of neurons in the thalamus relate to the motor cortex, iron deposition, as well as GM atrophy of the thalamus, may be attributed to the secondary effect after the impairment of neurons in the motor cortex. Since this study only offered a linear correlation between iron deposition and GM atrophy, it is, therefore, still a matter of debate whether iron deposition is a direct cause or a secondary event of structural impairment. The exact pathophysiological mechanism of ALS is multifactorial and requires further clarification.

The ALSFRS-R is a validated rating instrument for monitoring the progression of disability in patients with ALS, which correlates significantly with quality of life. The UMN score represents the severity of the UMN damage. In this study, we found that the QSM value of the precentral gyrus was significantly positively correlated with the UMN score, while the GM volume of the left precentral gyrus was negatively correlated with the UMN score. These observations indicate that iron deposition and GM atrophy in the precentral gyrus may together contribute to the UMN impairments. In addition to the precentral gyrus, the QSM value of the thalamus was significantly negatively correlated with the ALSFRS-R, suggesting that thalamic iron deposition is associated with ALS disease severity. The associations between thalamocortical iron deposition and the ALSFRS-R as well as the UMN score indicate strong clinical relevance. Specifically, iron deposition in the thalamus may affect the ability of sensorimotor information transmission and, therefore, patients with higher iron content in the thalamus exhibit more impaired overall physical functions as shown in the lower ALSFRS-R score. Based on these observations, we considered that the increased iron content in the thalamus may be an objective neuroimaging biomarker beyond the motor cortex that can reflect the disease severity of ALS. We did not obtain a significant correlation between the disease duration and the GM volume in the motor cortex. There are two potential reasons. First, the heterogeneity of the ALS in addition to the small sample size in this study may cause a negative finding. Second, we selected ROI in a *post-hoc* manner, which was based on the clusters that showed the most significant accumulation of iron content, which may not directly correlate most with the disease duration.

The major limitation of this study is the relatively small sample size, which limited the statistical power. As a result, the negative correlation between iron deposition and GM volume in the thalamus reached a one-tailed significance level. Although we have a prior hypothesis, the association between iron deposition and GM volume may become more significant with relatively larger sample size. In addition, although the QSM value correlates strongly with iron deposition in GM, it is an indirect measurement, which is difficult to mark the exact pathological process in the neurons. The third limitation of this study is the lack of postmortem examination so we cannot confirm the exact neurological underpinnings. From the methodological point of view, we applied the T1-normalization approach to normalizing the QSM images and found eight subjects that the images were not coregistered into the MNI template accurately. Future studies with a large sample size are encouraged to investigate a more appropriate approach for better normalization of the QSM images.

In conclusion, this study provided evidence of increased QSM values, decreased GM volume in the motor-thalamic loop in patients with ALS, supporting a probable association of iron deposition and GM atrophy as hallmarks of the disease. The current results may bring insights into the thalamic pathologic mechanism in ALS.

## Data Availability Statement

The original contributions presented in the study are included in the article/Supplementary Material, further inquiries can be directed to the corresponding author.

## Ethics Statement

The studies involving human participants were reviewed and approved by Xuanwu Hospital. The patients/participants provided their written informed consent to participate in this study.

## Author Contributions

QL contributed to the volunteer recruitment, experimental design, data collection, statistics, and manuscript preparation. WZ, XW, and YD contributed to the patient recruitment and clinical data collection. ZZ contributed to the data analysis and statistics. JL contributed to the conception, funding, study design, supervision, and manuscript preparation. All authors contributed to the article and approved the submitted version.

## Funding

This study was supported by the National Natural Science Foundation of China (82001352, 81801255), the Capital Health Development Research Project (2018-4-2015), the Beijing Hospitals Authority Youth Program (QML20190803), and the Beijing Municipal Administration of Hospitals' Ascent Plan, code: DFL20180802.

## Conflict of Interest

The authors declare that the research was conducted in the absence of any commercial or financial relationships that could be construed as a potential conflict of interest.

## Publisher's Note

All claims expressed in this article are solely those of the authors and do not necessarily represent those of their affiliated organizations, or those of the publisher, the editors and the reviewers. Any product that may be evaluated in this article, or claim that may be made by its manufacturer, is not guaranteed or endorsed by the publisher.
