# Supplementary material for: Beyond the Motor Cortex: Thalamic Iron Deposition Accounts for Disease Severity in Amyotrophic Lateral Sclerosis
Source: Front Neurol. 2022 Feb 24;13:791300. doi: 10.3389/fneur.2022.791300 (PMC8907117; doi:10.3389/fneur.2022.791300)

Supplementary Materials

Figure S1. Example of excluded subjects due to co-registration failure.

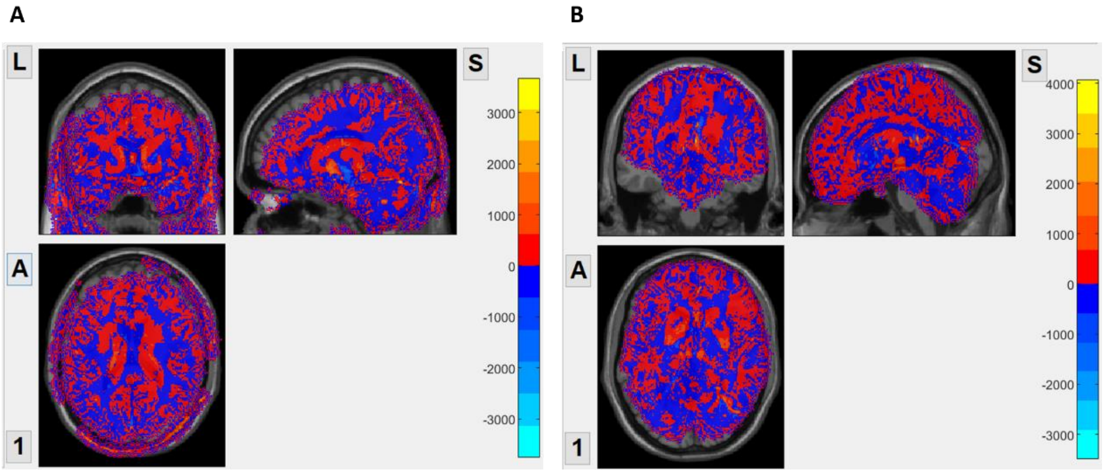

Panel A shows the mis-registration image from an ALS patient with significantly visible failure of registration in the frontal lobe. Panel B shows the mis-registration image from a NC where the occipital lobe as well as the bilateral medial temporal gyrus was not co-registered

Figure S2. Increased iron deposition in ALS patients with P < 0.005 uncorrected threshold

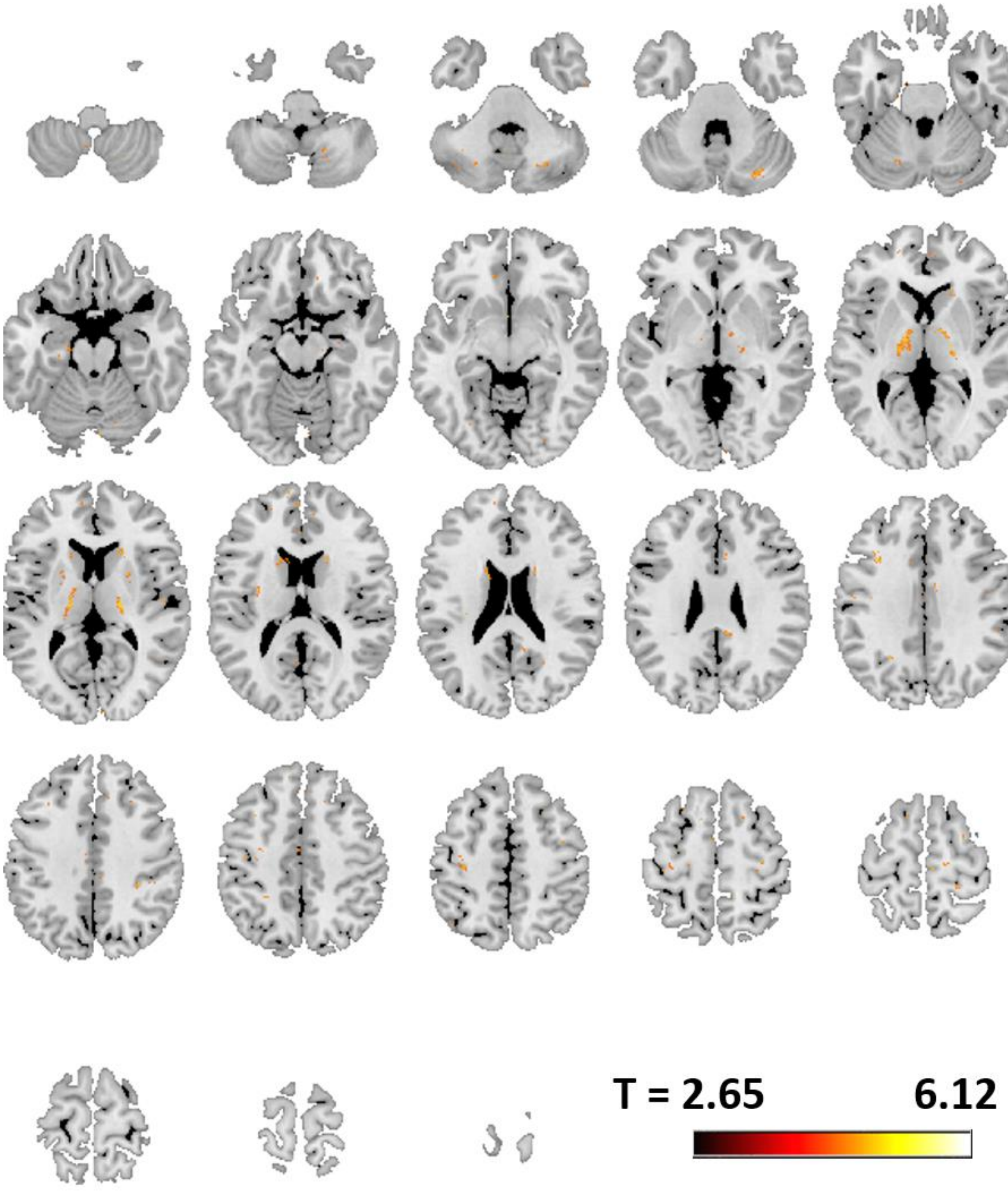

Figure S3 Decreased grey matter volume in ALS patients with P < 0.005 uncorrected threshold

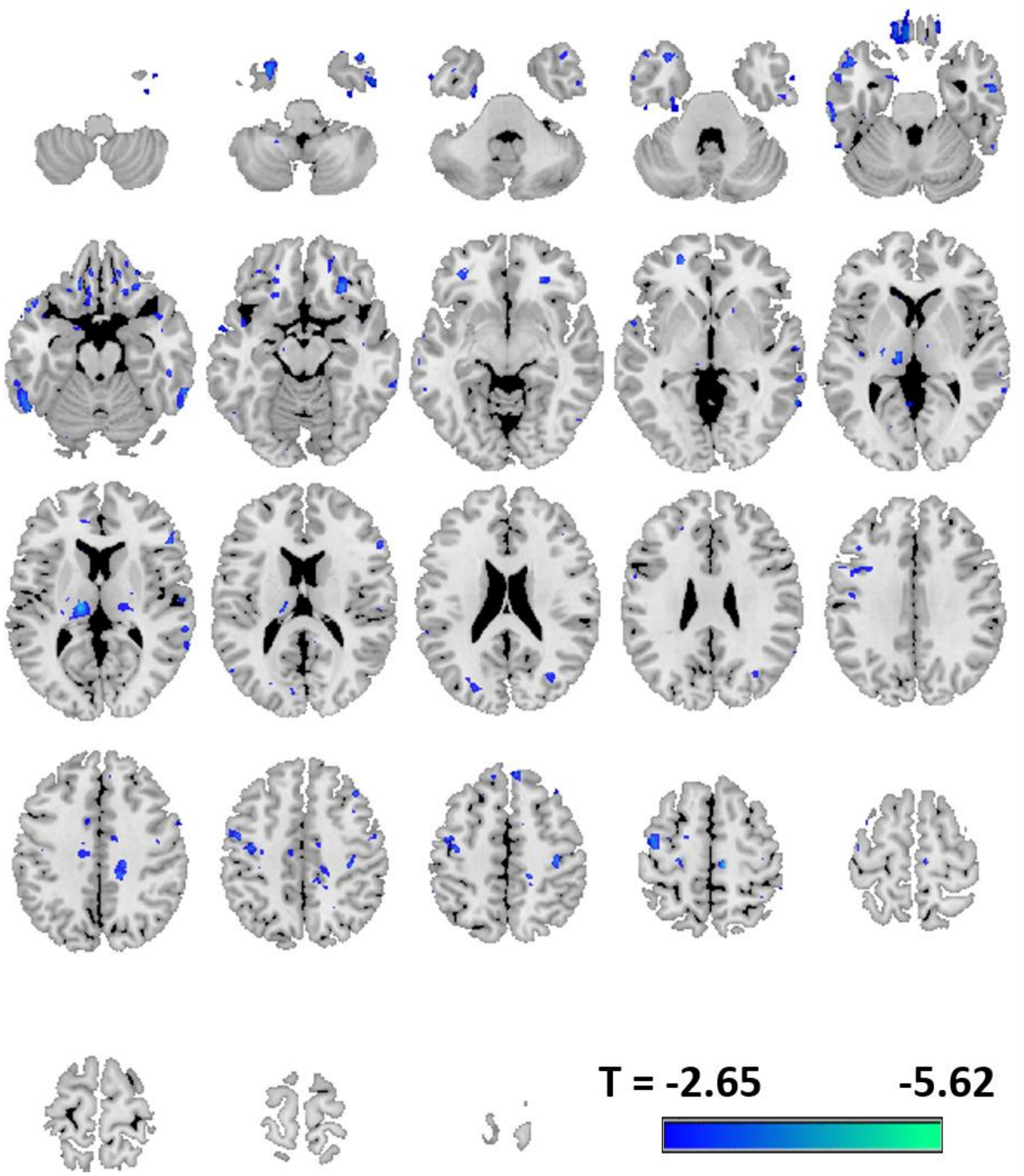

Supplement: Supplementary file 1 [file Image_1.pdf]
